# The impact of the COVID-19 pandemic on diagnostic procedures in hearing healthcare in Brazil: time series analysis and regional inequalities COVID-19 and hearing diagnostics in Brazil

**DOI:** 10.1016/j.bjorl.2026.101840

**Published:** 2026-05-28

**Authors:** Guilherme Corrêa Guimarães, Arthur Menino Castilho, Vagner Antônio Rodrigues da Silva

**Affiliations:** Universidade Estadual de Campinas (UNICAMP), Faculdade de Ciências Médicas, Department of Otorhinolaryngology, Head and Neck Surgery, São Paulo, SP, Brazil

**Keywords:** Hearing loss, COVID-19 pandemic, Delivery of health care, Public health systems research, Health care disparities

## Abstract

**Objective:**

This study aimed to evaluate the national and regional impact of the COVID-19 pandemic on diagnostic procedures in hearing healthcare in Brazil, highlighting temporal trends, regional disparities, and post-pandemic recovery.

**Methods:**

A retrospective, descriptive, ecological time series study was conducted using publicly available data from DATASUS, the national health database maintained by the Brazilian Ministry of Health. Data from January 2009 to December 2024 were analyzed, covering audiometry, newborn hearing screening, Otoacoustic Emissions (OAE), and Auditory Brainstem Response (ABR). Interrupted Time Series (ITS) and simple regression models were applied to assess changes across three periods: pre-pandemic, pandemic, and post-pandemic. Analyses were stratified by national level, Brazil’s five regions, and the state of São Paulo.

**Results:**

All major hearing diagnostic procedures experienced significant reductions at the onset of the pandemic. Audiometry volumes decreased by 18.5% nationally, with São Paulo showing the most pronounced decline (−26.3%). Newborn hearing screening remained stable during the pandemic but dropped by 15.4% in the post-pandemic period, while OAE (+31.1%) and ABR (+55.0%) eventually exceeded pre-pandemic levels. Recovery was heterogeneous: the North and Northeast showed proportionally greater rebounds, while Sao Paulo and the Central-West demonstrated limited recovery. Regional disparities were evident, with wealthier regions maintaining procedure volumes disproportionate to their population share.

**Conclusion:**

The pandemic caused marked disruptions in hearing healthcare delivery in Brazil, with heterogeneous recovery across regions. Vulnerable populations, particularly children and older adults, were disproportionately affected by delayed diagnoses and rehabilitation. While teleaudiology offered a partial solution during service suspensions, its effectiveness was constrained by structural and digital inequalities. Strengthening health system resilience will require prioritizing pediatric backlogs, integrating hearing health into healthy aging strategies, and embedding auditory care within broader frameworks to ensure continuity of essential services in future crises.

**Level of evidence (Oxford 2011):**

2b.

## Highlights

• COVID-19 sharply reduced hearing diagnostic procedures in March 2020.
• Recovery was uneven: North/Northeast rebounded; São Paulo lagged.
• Post-pandemic screening decline risks delayed pediatric diagnoses.
• OAE and ABR exceeded pre-pandemic levels, audiometry recovery modest.
• Regional inequities persisted, reflecting wealth and infrastructure gaps.

## Introduction

In late 2019, the emergence of SARS-CoV-2 led to the declaration of COVID-19 as a global pandemic in March 2020, resulting in unprecedented disruptions to healthcare services worldwide.^1^, ^2^, ^3^ These disruptions directly affected hearing healthcare through reduced outpatient capacity, suspension of elective services, and delays in diagnostic and rehabilitative pathways. Hearing healthcare was among the most impacted services.^1^, ^2^, ^3^ Hearing healthcare was among the most impacted services. These interruptions are particularly concerning for pediatric populations, as early detection of hearing loss and timely intervention are essential to ensure adequate auditory input during critical periods of neurodevelopment. This is crucial to optimize language acquisition, cognitive development, and social integration.^4^ At the same time, untreated hearing loss in adults and older individuals has been consistently associated with reduced social engagement, depression, and accelerated cognitive decline.^5^^,^^6^

Globally, the burden of hearing loss is increasing. According to the World Health Organization, more than 430 million people live with disabling hearing loss.^3^ These figures highlight the urgent need for resilient auditory healthcare systems. However, while the pandemic’s effects on hearing care have been documented in high-income settings such as the United States and the United Kingdom, evidence from low- and middle-income countries remains limited.^2^^,^^7^

Brazil represents a unique case study in this regard. As the largest country in Latin America, Brazil is characterized by profound regional heterogeneity in socioeconomic development, healthcare infrastructure, and accessibility of specialized services.^8^ The five major geographic regions ‒ North, Northeast, Central-West, Southeast, and South ‒ differ significantly in demographic density, distribution of healthcare facilities, and governmental investment. Additionally, the state of São Paulo alone accounts for a disproportionately high volume of specialized health procedures compared with entire regions of the country.^9^ Against this backdrop, analyzing the impact of COVID-19 on hearing diagnostic procedures in Brazil provides important insights into both the direct effects of the pandemic and the structural inequalities that predate it.

The aim of this study is therefore to evaluate national and regional trends in hearing diagnostic procedures in Brazil, assessing the effects of pandemic-related disruptions and post-pandemic recovery. These findings may help guide strategies to strengthen health system resilience and ensure continuity of hearing healthcare services in the post-pandemic period.

## Materials

### Study design

Retrospective, descriptive, and ecological study based on secondary data from Brazil’s national public health system. The primary aim was to assess the impact of the COVID-19 pandemic on hearing health services in the country, particularly focusing on diagnostic procedures.

### Data source

Data were obtained from the Department of Informatics of the Brazilian Unified Health System (DATASUS), an official national administrative database that provides publicly available information on healthcare procedures performed within the Unified Health System (SUS) across Brazil. DATASUS offers standardized, routinely collected data with nationwide coverage, enabling longitudinal analyses of procedure volumes and regional comparisons. All data analyzed in this study are publicly available through DATASUS (https://datasus.saude.gov.br/informacoes-de-saude-tabnet/).

### Study period

The study analyzed data from January 2009 to December 2024, divided into three timeframes: Pre-pandemic period from January 2009 to February 2020, Pandemic period from March 2020 to December 2021 and Post-pandemic period from January 2022 to December 2024.

### Inclusion criteria and variables

Procedures related to hearing diagnosis were grouped into the following categories: (i) Audiometric evaluations, including pure-tone audiometry, speech audiometry, free-field audiometry, and visual reinforcement audiometry; (ii) Electrophysiological tests, including Auditory Brainstem Response (ABR); (iii) Newborn hearing screening, including Otoacoustic Emissions (OAE) and ABR (test and retest); and (iv) Otoacoustic emission tests, including transient-evoked OAE and distortion-product OAE.

### Statistical analysis

Each procedure was organized by region, at the national level, and for the state of Sao Paulo. For each study period, monthly averages, percentage variations relative to the pre-pandemic baseline, and recovery ratios (post-pandemic/pre-pandemic) were calculated. To assess the proportional distribution of procedures, regional procedure rates were compared with the corresponding regional population across the three periods analyzed. Data from the state of São Paulo were analyzed separately, given that the state alone is larger and has a higher volume of procedures than entire Brazilian regions.

Time series analyses were performed using Interrupted Time Series (ITS) regression models and simple regression models. Outliers identified in the monthly volumes of certain procedures, which could disproportionately influence the results, were corrected by replacing their values with the average of the preceding and subsequent months, thereby maintaining the temporal consistency of the series. All analyses were conducted using R software, version 4.4.2.

### Ethical considerations

As this study used publicly available and anonymized secondary data, no approval from an ethics committee was required. The study was conducted in accordance with ethical standards for the use of public datasets.

### Study limitations

The use of administrative data may be subject to underreporting, coding errors, and lack of clinical granularity. However, the national coverage and longitudinal scope of DATASUS allow for meaningful population-level analysis. Despite the limitations, the study provides valuable insights into impact on the hearing evaluation during the COVID-19 pandemics and its consequences.

## Results

### Audiometric evaluations

At the national level, the ITS model indicated a significant upward trend of 361 procedures per month in the pre-pandemic period (p < 0.001). In March 2020, there was an abrupt reduction of 84,509 procedures compared with the pre-pandemic trend (p < 0.001), followed by a significant increase of 2,857 procedures per month during the pandemic (p = 0.001). In the post-pandemic period, no immediate significant change was observed, but a declining monthly trend of 2,358 procedures was identified (p = 0.007). The simple regression model confirmed a significant reduction during the pandemic (−23,477 procedures/month, p = 0.040) and a strong recovery post-pandemic (+23,209 procedures/month, p < 0.001).

**Fig. 1:**
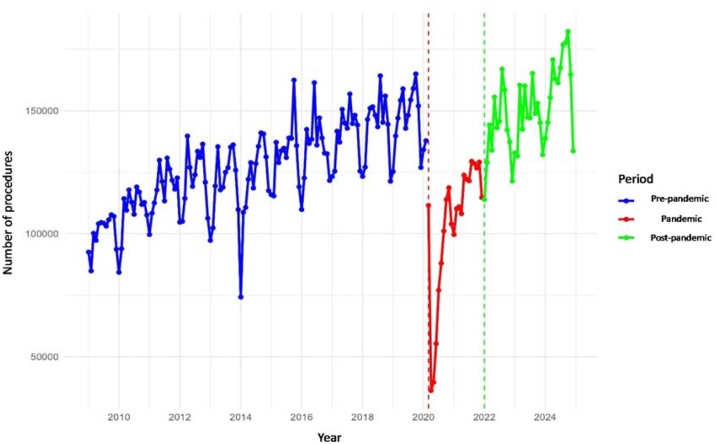
Monthly volume of audiometry procedures in Brazil.

Regional analyses revealed heterogeneous patterns. In the Central-West and Northeast, significant growth trends were observed before the pandemic, followed by sharp reductions at its onset (−4,744 and −19,154 procedures, respectively: both p < 0.001). Both regions showed a significant increase in monthly volumes during the pandemic, but a negative trend in the post-pandemic period (p < 0.001 for both). In the North, the ITS model detected a modest drop at the beginning of the pandemic (−1,142; p = 0.045), though monthly variations during and after the pandemic were not significant. All regions showed significant upward trends before the pandemic, sharp reductions in March 2020 (−15,030, −14,829, and −29,107 procedures, respectively; p < 0.001), and positive but temporary monthly increases during the pandemic (p ≤ 0.004). In the post-pandemic period, no significant immediate increases were detected, but São Paulo and the Southeast exhibited declining monthly trends (−817 and −341, respectively) (Table 1, Table 2, Fig. 1)

**Table 1:** Regional averages of audiometry procedures in the pre-pandemic, pandemic, and post-pandemic periods, with percentage changes and recovery ratios relative to pre-pandemic levels.

Region	Mean (Pre)	Mean (Pandemic)	Mean (Post)	Variation Pandemic vs. Pre (%)	Variation Post vs. Pre (%)	Recovery Ratio
Brazil	126,665	103,188	149,874	−18.5	18.3	1.18
Central-West	8,097	7,240	10,630	−10.6	31.3	1.31
Northeast	25,669	20,070	29,723	−21.8	15.8	1.16
North	5,945	7,221	8,879	21.5	49.3	1.49
Southeast^a^	22,436	17,074	26,283	−23.9	17.1	1.17
South	19,869	18,940	28,480	−4.7	43.3	1.43
São Paulo	44,316	32,644	45,879	−26.3	3.5	1.04

a São Paulo excluded from the Southeast values.

**Table 2:** Regional proportion of audiometry procedures compared to the population distribution across the pre-pandemic, pandemic, and post-pandemic periods.

Region	Pre-pandemic Procedures (%)	Pre-pandemic Population (%)	Pandemic Procedures (%)	Pandemic Population (%)	Post-pandemic Procedures (%)	Post-pandemic Population (%)
Central-West	6.4	7.5	7.0	7.8	7.1	9.8
Northeast	20.3	27.7	19.4	27.1	19.8	32.8
North	4.7	8.4	7.0	8.8	5.9	10.5
Southeast^a^	17.8	20.3	16.5	20.2	17.5	16.8
South	15.7	14.3	18.4	14.3	19.0	11.9
São Paulo	35.1	21.7	31.6	21.9	30.6	18.2

a São Paulo excluded from the Southeast values.

### Newborn hearing screening

**Fig. 2:**
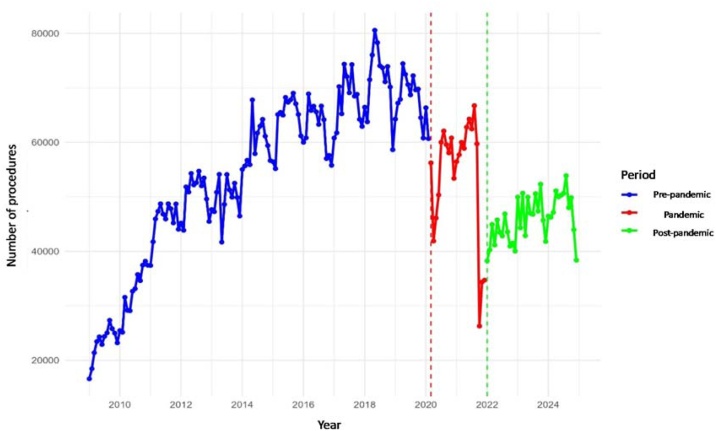
Monthly volume of the Newborn hearing screening procedure in Brazil.

Newborn hearing screening volumes remained stable nationwide during the pandemic (−0.2%) but decreased in the post-pandemic period (−15.4%), with a recovery ratio of 0.85. Among regions, the Central-West was the only one to exceed pre-pandemic levels, with increases during both the pandemic (+11.1%) and post-pandemic (+22.9%), resulting in a recovery ratio of 1.23. The Southeast (excluding São Paulo) also showed a marked increase during the pandemic (+26.3%) and partial recovery afterward (+16.7%; ratio = 1.17). In contrast, the North experienced the greatest impact, with sharp reductions during the pandemic (−26.1%) and further decline post-pandemic (−56.2%), yielding the lowest recovery ratio (0.44). The Northeast and South remained relatively stable during the pandemic but declined in the post-pandemic period (−18.0% and −14.2%, respectively). São Paulo recorded negative variations in both phases (−11.2% during the pandemic; −33.3% post-pandemic), with a recovery ratio of 0.67 (Table 3, Fig. 2).

**Table 3:** Regional averages of Newborn Hearing Screening in the pre-pandemic, pandemic, and post-pandemic periods, with percentage variation and recovery ratio in relation to the pre-pandemic level.

Region	Mean (Pre)	Mean (Pandemic)	Mean (Post)	Variation Pandemic vs. Pre (%)	Variation Post vs. Pre (%)	Recovery Ratio
Brazil	54,307	54,225	45,968	−0.2	−15.4	0.85
Central-West	3,738	4,153	4,593	11.1	22.9	1.23
Northeast	11,640	11,752	9,546	1.0	−18.0	0.82
North	5,893	4,354	2,581	−26.1	−56.2	0.44
Southeast^a^	8,993	11,361	10,491	26.3	16.7	1.17
South	14,666	14,572	12,581	−0.6	−14.2	0.86
São Paulo	9,258	8,223	6,177	−11.2	−33.3	0.67

a São Paulo excluded from the Southeast values.

Across the country, Newborn hearing screening increased significantly before the pandemic (+359 procedures/month, p < 0.001), but showed an abrupt drop in March 2020 (−19,966, p < 0.001) and a post-pandemic reduction of 8,339 procedures/month compared with pre-pandemic levels (p = 0.018). Regionally, the North recorded consistent losses, with a reduction of 2,611 procedures at the onset of the pandemic (p < 0.001), an additional decline in January 2022 (−1,836, p = 0.031), and lower averages during (−1,539/month, p = 0.001) and after the pandemic (−3,312/month, p < 0.001). The South also declined sharply in March 2020 (−4,630, p < 0.001), showed a negative monthly trend during the pandemic (−202/month, p = 0.022), and remained below pre-pandemic levels in the post period (−2,085/month, p = 0.023). São Paulo presented pre-pandemic growth (+31/month, p < 0.001), a sustained decline during the pandemic (−197/month, p = 0.001), and lower averages post-pandemic (−3,081/month, p < 0.001), despite a modest recovery trend (+176/month, p = 0.005). In contrast, the Southeast (excluding São Paulo) exhibited a strong pre-pandemic increase (+83/month, p < 0.001), a drop of 3,830 procedures in March 2020 (p < 0.001), but significantly higher monthly averages during the pandemic compared with pre-pandemic levels (+2,368/month, p = 0.019).

### Otoacoustic emissions

**Fig. 3:**
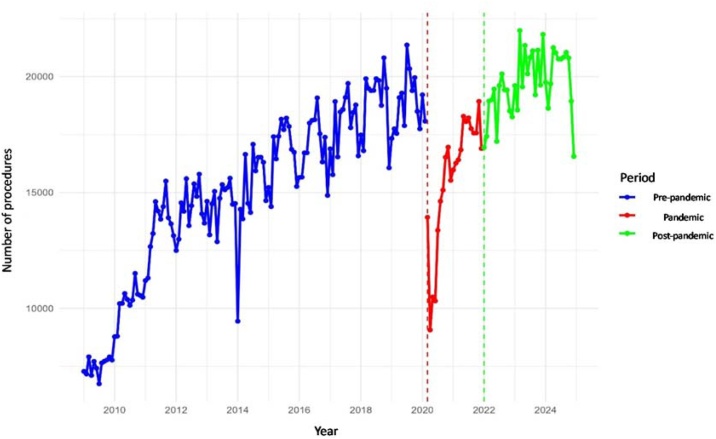
Monthly volume of the OAE procedure in Brazil.

At the national level, Otoacoustic Emission (OAE) procedures showed significant pre-pandemic growth (+84/month, p < 0.001), a sharp drop at the onset of the pandemic (−8,976, p < 0.001), recovery during the pandemic (+264/month, p < 0.001), and a subsequent decline post-pandemic (−296/month, p < 0.001), although average volumes remained higher than pre-pandemic (+4,674/month, p < 0.001) (Table 4, Fig. 3).

**Table 4:** Regional averages of the OAE procedure in the pre-pandemic, pandemic, and post-pandemic periods, with percentage variation and recovery ratio in relation to the pre-pandemic level.

Region	Mean (Pre)	Mean (Pandemic)	Mean (Post)	Variation Pandemic vs. Pre (%)	Variation Post vs. Pre (%)	Recovery Ratio
Brazil	15,028	15,671	19,702	4.3	31.1	1.31
Central-West	1,052	1,335	1,242	26.9	18.1	1.18
Northeast	7,294	7,378	9,157	1.1	25.5	1.26
North	1,618	2,363	3,496	46.0	116.0	2.16
Southeast^a^	2,163	1,845	2,214	−14.7	2.4	1.02
South	1,018	1,265	1,748	24.3	71.8	1.72
São Paulo	1,883	1,487	1,844	−21.1	−2.1	0.98

a São Paulo excluded from the Southeast values.

Across regions, we observed a consistent pattern of abrupt reductions in March 2020, followed by partial recovery during the pandemic and, in some regions, new declines after January 2022. In the Central-West, procedure volumes increased before the pandemic (+8 month) but dropped sharply in March 2020 (−647), followed by recovery during the pandemic (+28/month) and a further decrease in January 2022 (−535) with a subsequent downward post-pandemic trend (−32/month), despite higher post-pandemic averages (+190/month). In the Northeast, a strong pre-pandemic increase (+47/month) was interrupted by a marked decline in March 2020 (−4,815), with recovery during the pandemic (+109/month) but declining trends afterward (−119/month), although post-pandemic averages remained higher (+1,863/month). In the North, volumes increased before and during the pandemic (+7/month and +39/month, respectively) and rose in January 2022 (+755), but subsequently declined (−53/month); the simple model confirmed overall increases during and after the pandemic. Similar dynamics were observed in the Southeast (excluding São Paulo) and South, with pronounced declines at the pandemic onset and only partial recovery, followed by additional reductions in 2022. Finally, in São Paulo, procedures increased before and during the pandemic (+3/month and +37/month), but showed a significant decline in the post-pandemic period (−55/month). All reported effects were statistically significant (p < 0.05).

### Auditory Brainstem response

**Fig. 4:**
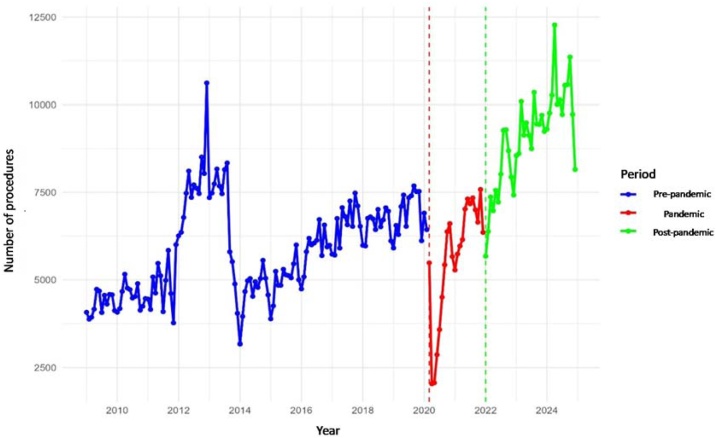
Monthly volume of the Auditory Brainstem Response (ABR) procedure in Brazil.

From a national perspective, ABR procedures showed pre-pandemic growth (+16/month), an abrupt decline in March 2020 (−3,627), a positive trend during the pandemic (+188/month), and a post-pandemic decline (−104/month), although average volumes remained higher than pre-pandemic levels (+3,211/month) (Table 5, Fig. 4).

**Table 5:** Regional averages of the Auditory Brainstem Response (ABR) procedure in the pre-pandemic, pandemic, and post-pandemic periods, with percentage variation and recovery ratio in relation to the pre-pandemic level.

Region	Mean (Pre)	Mean (Pandemic)	Mean (Post)	Variation Pandemic vs. Pre (%)	Variation Post vs. Pre (%)	Recovery Ratio
Brazil	5,833	5,645	9,044	−3.2	55.0	1.60
Central-West	601	482	539	−19.8	−10.4	0.90
Northeast	2,020	2,033	3,048	0.6	50.9	1.50
North	881	384	1,856	−56.4	111.0	2.10
Southeast^a^	732	1,044	1,210	42.7	65.4	1.70
South	416	723	1,220	74.0	193.0	2.90
São Paulo	1,187	979	1,170	−17.6	−1.4	1.00

a São Paulo excluded from the Southeast values.

Across regions, ABR procedures showed an abrupt decline in March 2020, followed by partial recovery during the pandemic and heterogeneous post-pandemic trajectories. Nationally, volumes increased before the pandemic (+16/month) but dropped sharply in March 2020 (−3,627), followed by a positive trend during the pandemic (+188/month) and a post-pandemic decline (−104/month), despite higher post-pandemic averages compared with pre-pandemic (+3,211/month). In the Central-West, volumes declined before the pandemic (−1/month) and decreased in March 2020 (−205), followed by recovery during the pandemic (+14/month) and an additional reduction in January 2022 (−124) with a sustained negative post-pandemic trend (−10/month). In the Northeast, pre-pandemic growth (+9/month) was interrupted by a sharp decline in March 2020 (−1,336) and followed by recovery during the pandemic (+55/month), with higher post-pandemic averages (+1,029/month). In the North, volumes declined pre-pandemic (−6/month) and dropped further in March 2020 (−291) but recovered during the pandemic (+23/month), increased in January 2022 (+470), and continued to rise post-pandemic (+27/month); the simple model confirmed a post-pandemic increase (+975/month). In the Southeast (excluding São Paulo), volumes increased pre-pandemic (+5/month), declined in March 2020 (−597), recovered during the pandemic (+45/month), but decreased again in January 2022 (−481) with a negative post-pandemic trend (−43/month). In the South, procedures increased pre-pandemic (+6/month), declined in March 2020 (−460), recovered during the pandemic (+28/month), but fell post-pandemic (−19/month), despite higher averages in the simple model after the pandemic (+804/month). Finally, in São Paulo, volumes increased before the pandemic (+3/month) but dropped substantially in March 2020 (−733), followed by partial recovery during the pandemic (+24/month) and a significant post-pandemic decline (−27/month). All reported effects were statistically significant (p < 0.05).

## Discussion

The present study provides a national, longitudinal assessment of hearing diagnostic procedures in Brazil, showing that key evaluations ‒ including pure-tone and speech audiometry, ABR, and newborn hearing screening ‒ experienced a marked reduction during the pandemic period. Given Brazil’s geographic and socioeconomic diversity, regional disparities across the pre-pandemic, pandemic, and post-pandemic periods are particularly relevant, as they may reflect not only the direct effects of COVID-19 but also the underlying heterogeneity of the national territory. The North and Northeast regions are generally more vulnerable, with limited healthcare resources and lower financial investment, whereas the Southeast and South tend to concentrate greater infrastructure and specialized services. The Central-West occupies an intermediate position, with a smaller population but better performance indicators than the North and Northeast.^10^^,^^11^ Importantly, these regional patterns are consistent with international evidence showing that pandemic-related disruptions disproportionately affected populations with greater structural vulnerability and reduced access to specialized care. Therefore, regional comparisons should be interpreted considering structural and financial inequalities, which translate into distinct healthcare realities within Brazil.^10^^,^^11^

### Impact on pediatric populations

Early identification of hearing loss is a cornerstone of child development, ensuring timely interventions that promote speech, language, cognitive, and social skills. The disruptions caused by the COVID-19 pandemic, including the suspension or delay of newborn hearing screening and diagnostic follow-up, jeopardized this critical timeline. In Brazil, our findings demonstrate significant reductions in neonatal screening volumes during and after the pandemic, particularly in the North and in São Paulo.

These findings are consistent with international reports. In the United Kingdom, parental surveys revealed distress and uncertainty due to postponed newborn hearing screenings during lockdowns.^12^ In the United States, Early Hearing Detection and Intervention (EHDI) benchmarks were missed in 2020, delaying diagnoses and rehabilitation pathways.^13^ European studies have also described interruptions to systematic neonatal screening.^7^ These delays are particularly concerning because they risk missing critical neuroplasticity windows, potentially leading to irreversible deficits in language and cognitive development.^4^^,^^14^ Priority should be given to addressing pediatric backlogs by actively identifying and screening children who missed early diagnostic opportunities, thereby reducing the risk of long-term developmental impairments.

### Impact on adults and older individuals

For adults and older populations, the consequences of disrupted auditory healthcare extend beyond diagnostic backlogs. Untreated hearing loss is strongly associated with social isolation, depression, reduced quality of life, and accelerated cognitive decline.^5^^,^^6^^,^^15^ During the pandemic, these vulnerabilities were amplified.

Mandatory lockdowns and the widespread use of face masks limited face-to-face communication and reduced access to lip-reading, a compensatory strategy for many individuals with hearing impairment.^16^ Consistent with this context, our national time-series analysis demonstrated an abrupt reduction in hearing diagnostic procedures at the onset of the pandemic (March 2020), followed by heterogeneous recovery patterns across regions during the pandemic and post-pandemic periods. Audiometric procedures and ABR showed marked declines, with only partial recovery in some regions and subsequent reductions in the post-pandemic period in certain settings, indicating persistent instability in access to diagnostic care.

For older adults, the loss of access to rehabilitative care likely exacerbated psychosocial distress. Longitudinal evidence demonstrates that untreated hearing loss increases the risk of dementia and accelerates cognitive.^6^^,^^17^ The pandemic may therefore have intensified trajectories of decline among older adults already vulnerable to isolation and sensory impairment.^18^

This context highlights the need to recognize hearing health as a determinant of healthy aging and mental well-being. Strengthening hearing care services for older adults, including strategies for maintaining communication during crises, must be a central component of public health resilience planning given its established association with social isolation, depression, and cognitive decline.

### Social and regional inequalities in access

Brazil’s regional disparities were clearly reflected in our results. While the Southeast and South concentrate infrastructure, financial resources, and specialized professionals, the North and Northeast face chronic underinvestment. São Paulo, analyzed separately due to its size and complexity, showed larger reductions than other regions and limited post-pandemic recovery.

These findings align with broader evidence. Historical analyses of the SUS indicate persistent inequalities in healthcare access, with poorer outcomes in regions of lower socioeconomic development.^10^^,^^11^ Studies also highlight that unmet health needs in Brazil disproportionately affect the North and Northeast, exacerbated by limited availability of specialized services.^8^^,^^19^ During the pandemic, these inequities were magnified, as resource reallocation further constrained access to non-urgent but essential care.

To mitigate regional inequalities, capacity-building strategies should prioritize strengthening outpatient diagnostic infrastructure in underserved regions, expanding the availability of trained professionals, and improving referral pathways to specialized services. In addition, targeted investment in decentralized service delivery and telehealth-supported follow-up may help maintain continuity of hearing care during future disruptions, particularly in regions with limited access to specialized centers.

### Teleaudiology: opportunities and barriers

Teleaudiology ‒ implemented within hybrid care models ‒ may strengthen hearing healthcare resilience by supporting follow-up and rehabilitation during future disruptions; however, its equitable adoption in Brazil will require addressing structural barriers such as limited internet access and digital literacy gaps, particularly in underserved regions and among older adults.^20^, ^21^, ^22^

## Conclusion

The COVID-19 pandemic had a profound impact on hearing healthcare delivery in Brazil, with marked reductions in essential procedures such as newborn hearing screenings, audiometry, and Brainstem Evoked Response Audiometry. These interruptions likely delayed diagnoses and rehabilitation, disproportionately affecting vulnerable populations such as children and older adults. The heterogeneous recovery patterns observed across regions highlight persistent structural inequalities and underscore the need to strengthen hearing healthcare capacity where access is most fragile.

Beyond Brazil, our findings align with global evidence that essential hearing services are highly vulnerable to system-wide shocks, reinforcing the importance of integrating hearing healthcare into public health resilience planning. Strengthening equitable diagnostic and rehabilitative pathways ‒ supported by capacity-building strategies and continuity-of-care models ‒ will be critical to reduce the long-term societal burden of hearing loss and to safeguard vulnerable populations during future crises.

## ORCID ID

Guilherme Correa Guimaraes: 0000-0002-8550-5635

Arthur Menino Castilho: 0000-0002-9024-8004

Vagner Antonio Rodrigues da Silva: 0000-0002-7335-4489

## Data availability statement

The authors declare that all data are available in repository.

## Declaration of competing interest

The authors declare no conflicts of interest.
